# On the social embeddedness of aeromobility: insights from the ecological self in intimate relationships

**DOI:** 10.1007/s43638-023-00064-4

**Published:** 2023-05-15

**Authors:** Paul S. Ruppel

**Affiliations:** 1 Faculty of Social Science, Social Psychology and Social Anthropology Section and Hans Kilian and Lotte Köhler Center for Cultural Psychology and Historical Anthropology, Ruhr University Bochum, Bochum, Germany; 2 Department of Applied Human Sciences, Magdeburg-Stendal University of Applied Sciences, Stendal, Germany; 3 Institute for Qualitative Research, International Academy Berlin, Berlin, Germany

**Keywords:** Climate change, Sustainability, Flying, Negotiation, Qualitative interview, Qualitative research

## Abstract

In this article the author addresses selected psychosocial aspects experienced, expressed, and negotiated by sustainability-oriented individuals who share an environmental awareness yet at the same time struggle to implement it in their everyday lives. Based on the analysis of interviews conducted with individuals and couples in Germany, the author focuses on individual and joint efforts to deal with ecological challenges in the face of aeromobility in times of climate change. The results indicate the advantage of conceptualizing aeromobility as a sometimes deeply socially embedded practice and thus constitutive reference point for the ecological self in relationships.

## Introduction

In recent years we have been witnessing a growing concern for environmental issues in different groups and (sub-)cultures. The *Fridays for Future* demonstrations are currently the most visible public signs of this concern, and there are several more such as *Extinction Rebellion* or *Stay Grounded*. Unfolding discourses and practices have allowed for the emergence of ecology as a form of subjectivation (Ruppel and Straub 2017). The ecological self cares for the environment, it cherishes ecological moral values and frequently regards them as moral universals. However, these moral orientations are not universally shared, not even by all members of one’s relevant ingroups or intimate partners. Furthermore, they are fluid—or culturally and biographically “polyvalent,” as Boesch put it (Straub et al. 2020)—serving different functions for different people in different situations.

In this article I outline insights I gained while studying the empirical field of (non-)flying sustainability-oriented individuals from a cultural psychological perspective.

I concentrate on psychosocial phenomena intertwined with action, inaction and negotiations about the usage, avoidance, or rejection of aeromobility within relationships in particular. I therefore address how the ecological self leads a life with his or her partner, sharing some norms, moral values, narratives, and attitudes but holding differing and sometimes irreconcilable viewpoints at the same time. Simultaneously, I intend to contribute to an interdisciplinary perspective of tourist flights in times of climate change.

Based on the interpretative analysis of selected excerpts from interviews with individuals and couples and with a focus on the ecological self in intimate relationships, I exemplify two insights relevant to the entire field.

First, aeromobility can be conceptualized as a socially embedded practice rather than as an individual behavior.

Second, for environmentally conscious individuals bridging the gap between moral convictions and actions is certainly an important topic. However, it is not only about action and inaction or bridging what is commonly known as the attitude–behavior gap. The ecological self is a relational self that must handle the feeling of ambivalence without necessarily eradicating it because even if individuals reduce or stop flying, they might still be confronted with the feeling of ambivalence in their partnerships. Hence, sometimes it is not so much about bridging an intraindividual gap but rather an interindividual gap, and seeking a common language and understanding to indicate or assert shared moral values while leading a life together.

## Emerging discourse on the environmental consequences of aeromobility

From a long-term perspective—the COVID-19 pandemic certainly constituting a temporary exception and a major setback to the industry—air transport service is a global growth market. More and more people from different national origins and social backgrounds choose the airplane as a means of transportation. The increase in aeromobility has attracted growing public (and academic) attention to the role of air traffic in the climate change debate.

From an environmental point of view, flying represents a particularly problematic mode of mobility owing to its comparatively high carbon dioxide emissions and other effects accelerating global climate change. It constitutes one of the environmentally most adverse human activities. Furthermore, it is a mode of travelling that is particularly difficult to organize in an environmentally friendly way. From this perspective, changing flying habits is considered an important step towards a less environmentally harmful lifestyle.

There are numerous areas in which environmental discourse has been lively: housing, heating, cooling, lighting, food, clothing, electronic consumer goods, mobility, to name just a few. Considering its quantitative significance (CO_2_ equivalents), the discourse on the environmental consequences of aeromobility emerged comparatively late. That, incidentally, shows that scientific findings and their communication certainly play a pivotal role when it comes to deciding which area to focus on. Yet, whether an area becomes ecologically and therefore morally salient is not a purely rational matter and depends on socio-cultural factors as well. Meanwhile, however, aeromobility has become an integral part of science-related and normative environmental discourse.

We also see that environmental and social concerns have started mingling. Although specific groups and people are particularly affected by the environmental consequences of growing air traffic, not everybody can afford or make use of this mode of transportation. Thus, some people with easy access to air travel began voicing a feeling of guilt. The public discourse terminology was even enriched by media reports on the Swedish neologism “Flygskam,” denoting the feeling of shame when flying, adding another term to what Harré et al. (1999) call “Greenspeak.”

The debate about the downsides of flying addresses not just the environmental, social, and individual but also political and economic implications of air traffic. The global network “STAY GROUNDED” (https://stay-grounded.org), which was formed in 2016 to organize action against the environmentally harmful effects of aviation, shows, as one of many examples, by its diverse actions, that the complexity of the topic makes it almost impossible to consider these various aspects independently of each other.

Although media (and academic) reports on the ecological effects of flying as well as the debate on individual responsibility sound unmistakably loud and clear, this is accompanied by background noises that reflect social, political, economic, and infrastructural questions. Flying has thus become a heavily contested practice and a moral issue. It is not merely related to exotic destinations, adventure, and prestige anymore but additionally to toxic emissions, environmental destruction, and an irresponsible burden for future generations. Within this altered framework of meaning, flying has become another ambivalent human activity, a lifestyle cherished and regretted at once. Furthermore, it has become part of an environmental discourse, where natural sciences and rationalistic perspectives exist side by side and blur with moral and moralizing viewpoints.

## Shifting from the individual to the social

Before turning to the empirical approach and analysis in the next sections, I briefly outline a theoretical rationale for a shift in focus from the individual to the social.

There is a tendency to discuss aeromobility on the level of the single person (for more on the individualization of ecological, ethical, and moral responsibility see Maniates 2001). From this perspective, to fly or to avoid flying appears to be an individual choice. Assumed freedom of choice allows for ascribing responsibility. This responsibility in turn is a central reference point in environmental discourse. It also provides the basis for moralizing discourse, where individuals are asked to justify their aeromobility, assuming that only the change within individual behavior and actions can help to solve the environmental crisis.

The focus on the single person is also reflected in the natural sciences or quantifying perspectives. In environmental discourse, unintended climate effects caused by flights are frequently quantified and symbolized or illustrated as a carbon footprint that can even be calculated for single individuals. This quantification is a highly de- and recontextualizing cultural practice based on comparisons.

A focus on the individual is certainly important, but it runs the risk of overestimating the impact the individual can have as well as the individual’s supposed freedom of choice, thus neglecting the social embeddedness of flying.

Therefore, shifting the perspective from flying and related reflections as mere individual decision making toward flying as a social practice helps to uncover its interpersonal and interactional dimensions, i.e., to view the ecological self as a self in an (intimate) relationship—and a “relational self” (Gergen 1991, 1994, 2009). Related concepts refer to a “dialogical self” (Hermans and Kempen 1993) and “narrative identity” (Brockmeier and Carbaugh 2001). This cultural psychological perspective, inspired by symbolic interactionism and social constructionism, sheds light on the struggle people undergo when trying to arrange their life in accordance with their own ecological and moral convictions as well as those of significant others, especially their intimate partners. Focusing on these psychosocial aspects enables us to analyze how individuals negotiate felt support or lack of understanding, the aspiration of sameness and consistency, and the feeling of inconsistency or ambivalence on the personal and interpersonal level; it allows us to get a better understanding of their coping with the lack or surplus of sameness and difference experienced.

## Empirical approach

The natural sciences provide us with a quantitative assessment of the environmental consequences of flying, e.g., by calculating air travel-related emissions. I chose a qualitative approach using interviews to explore the psychosocial phenomena related to aeromobility in times of climate change. Qualitative interviews allow for the study of personal experiences, subjective perspectives, and meaning-making processes, and the way in which they are articulated and negotiated in a relatively open manner. This makes interviews particularly suitable for exploring the complexities in this emerging field. My empirical basis consists of interviews with individuals (Witzel and Reiter 2012) and couples (Wimbauer and Motakef 2017) who aspire to live sustainability-oriented lifestyles. Some of them refrain from tourism-related air travel for reasons of climate protection. Some consider refraining from flying in the future. Some like the idea of less air traffic and less pollution of the planet but still stick to flying.

In analyzing the autobiographical narratives and accounts, I used approaches borrowed from grounded theory methodology (for a short overview see Ruppel and Mey 2017) and inspired by relational hermeneutics (Straub 2006). Based on the sequential analysis of single cases and the establishment of conceptual ideas across cases, I provide preliminary conceptualizations and answers to the question how sustainability-oriented individuals cope with the supposed contradiction between a heightened awareness of climate change and increasing aeromobility. In short: *How do individuals and couples handle their conflicting perspectives about flying on a psychological and social level and what happens when they stop flying?*

### Sample

With traditional psychological concepts such as cognitive dissonance (Festinger et al. 1956) and the attitude–behavior gap (LaPiere 1934) in mind, I started out interviewing individuals who consider it harmful for the environment to fly but still take the airplane. These individuals appeared to view the protection of the environment—at least partly—as their personal responsibility and not solely that of companies or governments. Analyzing their accounts, I was intrigued by their ability to tolerate ambiguity^1^ and ambivalence without necessarily trying to eradicate it. Ambivalence and polyvalence became relevant sensitizing concepts (Blumer 1954). Interested to learn in what ways individuals who stopped flying owing to environmental considerations might also experience ambivalence, I decided to include them in the sample. Analyzing their interviews made me aware that the ambivalence does not necessarily vanish when individuals stop flying. I came to understand that ambivalence does not need to relate only on one’s own action and inaction but can also be linked to the action and inaction of others, especially significant others such as family members, friends, and partners. This sampling strategy finally led me to empirically acknowledge the important role of the social dimension of aeromobility in times of climate change, the social embeddedness of the actors, and its influence on their action and inaction. This insight encouraged me to expand the sample further and include interviews with couples who fly or do not fly to better understand the social dimension and explore how flying can be both a challenge for the environment and intimate relationships as well.

## Empirical insights into the social embeddedness of aeromobility

In this section I trace the social embeddedness of aeromobility. I do so by discussing some selected results gained during the research process as this field was just emerging. The focus lies on flying in the context of intimate relationships, as reflected upon by individuals and couples.

### In search for sameness in relationships

Even when holding strong individualistic convictions and seeing oneself as tolerant, with no ambition to dictate how others shall live their lives, the ecological self in relationships might aspire, desire, wish, or require its partner to share its concern for the environment. Living together while not sharing ecological moral values can challenge the assumed or desired moral unity as well as the aspiration to sameness. It might become questionable if common perceptions, common visions, and narratives of the past, the present, and the future exist. This can—as exemplified in the following excerpt—produce difference or allow it to surface.

(Excerpt from an interview with Bea)^2^

My boyfriend is a business consultant and the kind of guy who just flies like crazy. So, all he actually does is flying, yes. And that’s just the way it is with me, that I somehow, I mean, he, he, he knows, too, that I just think about things like that, and that I’ll clearly say: “One shall not fly to Cologne and certainly not to Frankfurt” and such. But somehow it is like this, so there somehow is just a total dividing line between us. That, that, that he does all that and that I know that, too, and that somehow is his job, but that I think it’s pretty blatant and outrageous, yes, if I think about it. (Bea, lines 239–247)

Here, the interviewee, Bea, characterizes her boyfriend as a person who flies extraordinarily frequently. She portrays him as a prototypical or stereotypical ecological anti-hero—a frequent flyer whose work seems to consist of flying back and forth. Bea highlights a contrast between him and herself by ascribing to herself a more intensive preoccupation with ecological issues. Her attitudes and reflections are said to be known to him and so is her normative stance, which strictly opposes (national) short-haul flights (given the existence of alternative means of traveling, for example, high-speed railway connections between cities such as Cologne and Frankfurt and their hometown). We see that Bea assumes shared knowledge about this topic and clearly expresses her stance also in direct speech—demanding herself and her partner to adhere to certain norms by avoiding short-haul flights. She seems willing to stand up to him and enforce her view so that on the level of action, at least some degree of similarity would be ensured. Bea articulates a strong feeling of difference in her relationship. She uses the metaphor of the “dividing line” (Bea, line 244) as given. Bea uses the present tense and stresses contrary actions and opposing stances within a relationship that is presented as static and stagnant in this regard.

Bea’s boyfriend does not stick to her norm. She knows that flying is part of his job. From Bea’s perspective, his flights are not legitimized as a vocational necessity. They rather serve as a basis for blaming him and criticizing him as a person, as she does not distinguish between the role of the flying consultant and other roles of her boyfriend. By doing this, she questions the integrity of her partner—at least regarding this context, which is the only context she has referred to so far. We see a transformation here. The initially ecologically framed problem (of intensive or excessive) aeromobility proves to be (also) a relationship problem. The experienced difference is rooted in perceived contrasting values and ways of acting. Moreover, it expresses itself in non-identification or distancing. The idea of a shared value orientation in the partnership is disappointed because of the boyfriend’s nonsustainability-oriented mobility practice. The different value orientations and their practical realization emerge—and they emerge unmasked (partly as a re-enactment during the interview). In the dyad divergent (and at least for the one person morally charged) self-expectations and expectations how the other should think and act come to the fore. The experience of difference is an experience of more or less separate moral worlds. The partner’s world is depicted in terms of a moral devaluation and is being reduced to one negatively connoted aspect of life. It must remain open to what extent the devaluation of the other can be seen as a means of improving the image of herself. Maybe it is rather a way of coping with an affront. Bea’s own strong values (it appears evident that this is not about mere taste preferences of minor importance to the person, but about fundamental convictions and orientations) are not only not shared by the partner, but continuously violated by practical action—the reference to ecological norms and morals as a stabilizing regulator in partnerships fails in this regard. He does not seem to care so much about what is important to her and does not comply with the demand for moral consideration and adaptation. Bea’s case clearly illustrates the aspect of aspiration to sameness in relationships and the futile search for it.

The next excerpt, taken from a different interview, is also an example of experienced lack of sameness. Different than Bea’s case, however, it shows the coupling of the aspiration of sameness with the expectation of consistency very explicitly.

(Excerpt from an interview with Clara)

My boyfriend flew twice or thrice, I think. In recent years. Once to Japan. Because of the study of architecture somehow. But there was also a lot of personal displeasure from me, too. That I maltreated him very, very much afterwards. And then, I think, two vacation trips. One to Gran Canaria and one to Fuerteventura or something. And those were things that were completely incomprehensible to me. And it was an issue even weeks afterwards. But as I said, there are several aspects to it. So, there was flying, that I did not understand, because my boyfriend is also vegan and, so, went along with many things. And I always thought we were very united in many things. So, in this whole world view, from which all actions somehow derive, I had the impression, we would be very similar. (Clara, lines 964–974)

Clara’s case vividly shows the expectation of consistency from oneself, from the other, and between each other (for a detailed single case analysis see Ruppel and Straub 2017). Put simply: it is about doing the same things for the same reason. The case demonstrates that what is at stake might be even more than shared ecological moral values and the same action or inaction, namely shared meaning-making (for the often-repeated call for attention to be given to meaning-making in psychological research see Bruner 1990).

Furthermore, both cases exemplify how a felt lack of sameness can be accompanied by a shift from an environmental to a relationship issue where the hope for a better world can take the shape of a moral battlefield in which partners struggle for power.

### Balancing in relationships—Calculating and compensating the emissions for another

The following excerpt of an interview with a couple exemplifies aspects of the phenomenon of balancing in relationships in times of climate change and how rationalistic and moral viewpoints mingle in a complex relationship arrangement.

(Excerpt from an interview with Xena and Tom)^3^

Tom: What was interesting, is that I um, already against the background of knowing, that we had spotted Vietnam as a holiday destination now for this summer, um Xena for the birthday, yes birthday it was, um such a, what’s it called?

Xena: Er you compensated the CO_2_ emissions for me. //(incompr.), yes.//

Tom: //Exactly, the compensations// I gave as a present.

Xena: 2500 kg CO_2_ compensated!

Tom: //Exactly.//

Interviewer 1: //(laughs)//

Tom: And that is, so that is interesting, because I have once, there I have myself, no idea, of course to me the comparison to a letter of indulgence,

Interviewer 1: (laughs)

Tom: um or where I just such an exculpating oneself again financially, is absolutely clear, and um that really always stimulates discussion, when you tell that. “Yes, what did you give your girlfriend as a present?” (Xena & Tom, lines 403–423)

In a co-constructive effort, the couple comes to speak about quite an unusual birthday gift—the compensation for the flight of the recipient. The gift and Xena’s explanation “you compensated the CO_2_ emissions for me” (Tom & Xena, line 407) is founded on the assumption that calculating environmental effects of flying on the individual level is possible and mechanisms for compensation exist that are quantifiable. These ways of compensating are not linked to individuals and their action, but compensation is possible for another person—a “personalized” gift. The amount of CO_2_ that is being compensated is linked to personal action by framing it as a gift related to a planned return flight from Vietnam. Although not flying can be seen as preemptive action against future consequences of climate change, here we see Tom acting in a preemptive manner by compensating for a flight that he assumes will be taken by his girlfriend in the future.

The impression that we are dealing with a purely goal-oriented, rational, and quantified concept of environment-oriented action changes when we consider that Tom immediately introduced the term “letter of indulgence” (Tom & Xena, line 417). It questions the assumption of Tom just calculating and compensating for the emissions for Xena. He presents himself as a person aware of a moral and ambivalent dimension specific to the subject matter: paying money to prevent the feeling of guilt. Later in the interview, we learn that Xena did not oppose flying to Vietnam. It was Tom pushing towards alternative modes of transportation. Tom also remarks that they did not calculate if the carbon footprint of the 4‑week train ride (for their outward journey they had envisioned taking the train) is actually better than flying. Xena insists, “It must be better” (Tom & Xena, line 599)—pointing at an ambivalent combination of rational calculation on the one hand and emotional attachment to supposedly environmental action on the other.

The possible recipient of the gift is hard to identify. In addition to the two connotations of a quantified way of compensating and a moral/immoral way of coping with bad feelings, there is a third dimension to it. The gift is something that is being talked about in the broader social network. Thus, it becomes part of the environmental discourse, the same way in which it now becomes part of academic discourse on this very contemporary issue of flying in times of climate change.

## Conclusion

In this article I outlined that flying has become an environmental and moral issue. With a focus on the ecological self in relationships, I pointed out a few selected psychosocial aspects traceable in the data that vaguely imply the complexities in this field: the social embeddedness, the nuanced ways of coping with ambivalence, and the blurring of rational, emotional, and moral perspectives. There are many more fascinating aspects related to it that were merely hinted at, e.g., self-presentation, self-optimization, and moralizing in this context, to name but a few. Finally, the focus can be widened and entail not only intimate partners but also family, friends, colleagues, and other social relations. As complex temporality is usually involved when it comes to climate change it is worthwhile also examining social relations beyond the here and now and investigate, for example, the interplay between considering future children or generations and environmental concern and action.

## Footnotes

1 For seminal work on the construct of ambiguity tolerance–intolerance see Frenkel-Brunswik (1949); for the systematization of attributes of intolerance of ambiguity see Bochner, who mentions characteristics such as the “need for certainty,” the “inability to allow for the co-existence of positive and negative features in the same object,” or “the early selection and maintenance of one solution in a perceptually ambiguous situation” (Bochner 1965, p. 394).

2 All names of the interviewees are pseudonyms. The interviews were conducted in German. The translation was done by me trying to retain the peculiarities common to spoken language.

3 The interview was conducted by Christopher Graetz and Philipp Stein, two former master’s students of mine.
